# The complete mitochondrial genome of *Pseudocellus pearsei *(Chelicerata: Ricinulei) and a comparison of mitochondrial gene rearrangements in Arachnida

**DOI:** 10.1186/1471-2164-8-386

**Published:** 2007-10-25

**Authors:** Kathrin Fahrein, Giovanni Talarico, Anke Braband, Lars Podsiadlowski

**Affiliations:** 1 Institut für Biologie, Freie Universität Berlin, Germany; 2 Zoologisches Institut und Museum, Ernst-Moritz-Arndt Universität Greifswald, Germany; 3 Institut für Biologie, Humboldt-Universität Berlin, Germany

## Abstract

**Background:**

Mitochondrial genomes are widely utilized for phylogenetic and population genetic analyses among animals. In addition to sequence data the mitochondrial gene order and RNA secondary structure data are used in phylogenetic analyses. Arachnid phylogeny is still highly debated and there is a lack of sufficient sequence data for many taxa. Ricinulei (hooded tickspiders) are a morphologically distinct clade of arachnids with uncertain phylogenetic affinities.

**Results:**

The first complete mitochondrial DNA genome of a member of the Ricinulei, *Pseudocellus pearsei *(Arachnida: Ricinulei) was sequenced using a PCR-based approach. The mitochondrial genome is a typical circular duplex DNA molecule with a size of 15,099 bp, showing the complete set of genes usually present in bilaterian mitochondrial genomes. Five tRNA genes (*trnW*, *trnY*, *trnN*, *trnL*(CUN), *trnV*) show different relative positions compared to other Chelicerata (e.g. *Limulus polyphemus*, *Ixodes *spp.). We propose that two events led to this derived gene order: (1) a tandem duplication followed by random deletion and (2) an independent translocation of *trnN*. Most of the inferred tRNA secondary structures show the common cloverleaf pattern except tRNA-Glu where the TψC-arm is missing. In phylogenetic analyses (maximum likelihood, maximum parsimony, Bayesian inference) using concatenated amino acid and nucleotide sequences of protein-coding genes the basal relationships of arachnid orders remain unresolved.

**Conclusion:**

Phylogenetic analyses (ML, MP, BI) of arachnid mitochondrial genomes fail to resolve interordinal relationships of Arachnida and remain in a preliminary stage because there is still a lack of mitogenomic data from important taxa such as Opiliones and Pseudoscorpiones. Gene order varies considerably within Arachnida – only eight out of 23 species have retained the putative arthropod ground pattern. Some gene order changes are valuable characters in phylogenetic analysis of intraordinal relationships, e.g. in Acari.

## Background

Due to their bacterial origin [1,2] mitochondria have retained a circular DNA double-helix, which in animals is sized between 12–30 kb. This is only a small part of the original bacterial chromosome, the majority was eliminated or transferred to the nucleus [3]. The mitochondrial DNA of Bilateria typically contains 37 genes and one AT-rich non-coding part, which putatively bears regulatory elements for transcription and translation and is therefore referred to as the mitochondrial control region [4]. In general the genes encode 13 protein subunits necessary for oxidative phosphorylation (*atp6+8*, *cob*, *cox1–3*, *nad1–6 *and *nad4L*), 22 transfer RNAs and two rRNAs (*rrnS *and *rrnL*) [5]. Except for the control region, mtDNA possesses only few non-coding sections between genes, even gene overlaps are common. E.g., in many species the last seven nucleotides of *atp8 *are also the first seven nucleotides of *atp6*. A similar overlap is often seen on the boundary between *nad4 *and *nad4L*. As a consequence, rearrangements in mitochondrial genomes most often disrupt genes and thus are deleterious – a possible reason for the stability of mitochondrial gene order [4].

Mitochondrial genomes have been proven useful for phylogenetic analyses [4]. Nucleotide or amino acid sequences as well as rearrangements in mitochondrial gene order are used as phylogenetic markers [6,7]. Gene rearrangements are considered to be valuable characters, because it is very unlikely that closely related taxa exhibit homoplastic translocations [8]. In addition the secondary structure of encoded tRNAs [9] and changes in the mitochondrial genetic code [10] have also been used as characters in phylogenetic analysis.

Ricinulei (hooded tickspiders) are a small order of arachnids, comprising 3 genera with 55 described species [11-14]. They are predatory animals that live in humid caves or leaf litter of tropical regions [15-17]. Species of *Ricinoides *occur in West Africa whereas species of *Cryptocellus *and *Pseudocellus *live in Central and South America [13]. Ricinuleids have body lengths of 3 to 10 mm [18] and their cuticle is strongly sclerotized and extraordinarily thick [19]. Several peculiarities characterize ricinuleids – a moveable hood (cucullus) in front of the prosoma covering the mouthparts, two jointed chelicerae, chelate pedipalps, elongated second legs, a tarsal copulatory organ on the third pair of legs of adult males, a locking mechanism between pro- and opisthosoma, which can be unlocked during mating and egg-laying, a 6-legged larvae, the lack of distinct eyes, and tracheal respiration [20-25]. According to morphological studies and some combined morphological and molecular analyses, ricinuleids are often considered to be the sister group of Acari [26-29], together forming the Acaromorpha. This clade is characterized by a unique post-embryonic development: a hexapodal larva followed by three octapod nymphal instars [28]. A gnathosoma with medially fused palpal coxae is another unique character of Acaromporpha. However the definition of a "gnathosoma" varies and its presence in Ricinulei is questioned by some authors [e.g. [30]]. The analysis of Shultz [27] obtained seven additional "homoplasious synapomorphies" supporting Acaromorpha (= these characters are not exclusively found in Acari and Ricinulei). Van der Hammen [31,32] however has questioned the monophyly of Acari and placed Ricinulei within the Acari as the sister group to Anactinotrichida (Parasitiformes s. str. + Opilioacariformes) [33]. Dunlop [34] suggested Ricinulei as sister group to the extinct Trigonotarbida which are collectively the sister of the extant Tetrapulmonata (Araneae, Uropygi, Amblypygi).

Because available sequence data from nuclear or mitochondrial genes is very limited for Ricinulei, recent molecular studies of arthropod phylogeny seldom included ricinuleids [e.g. [35]]. With a combined data set (93 morphological characters and 18S + 28S rRNA sequences) Wheeler & Hayashi [36] placed Ricinulei as sister group to Acari. In another combined analysis by Giribet et al. (253 morphological characters, 18S and 28S rRNA, [37]) Ricinulei appear as the sister group of Tetrapulmonata (Araneae + Amblypygi + Uropygi). When fossil taxa were included, a close relationship between the fossil Trigonotarbida and Ricinulei was recovered and both together were the sister group to Tetrapulmonata. This phylogenetic hypothesis corresponds with that of Dunlop [34], but Giribet et al. mentioned the instability of this relationship: In their analysis of only the molecular data set Ricinulei is early branching within the arachnid tree as the sister group to all remaining arachnids [37].

In this study, we present the first complete mitochondrial genome of a member of Ricinulei, the hooded tickspider *Pseudocellus pearsei *(Chamberlin & Ivie, 1938). The sequence data is used to unveil phylogenetic relationships between Arachnida. Furthermore, the gene order of mitochondrial genomes from all available arachnid species is compared in order to reconstruct the events leading to derived genome arrangements and to evaluate the phylogenetic significance of gene translocations within the Arachnida.

## Results and Discussion

### Genome organization, gene order and non-coding parts

**Figure 1:**
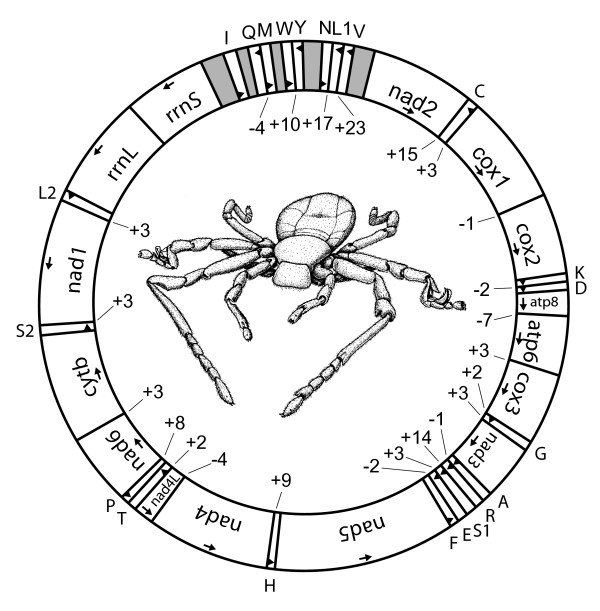
Mitochondrial genome map of *P. pearsei*. Transfer RNA genes are depicted by their one letter amino acid code (L_1_: *trnL*(CUN), L_2_: *trnL*(UUR), S_1_: *trnS*(AGN), S_2_: *trnS*(UCN)). Numbers indicate non-coding nucleotide spacers between genes (positive values) or gene overlap (negative values). Arrows indicate orientation on (+)strand (clockwise) or (-)strand (counterclockwise). Grey shaded parts represent larger non-coding regions (>50 bp). Line drawing of *P. pearsei *by Peter Adam. Body lengths of the animal is 4.6 mm.

**Figure 2:**
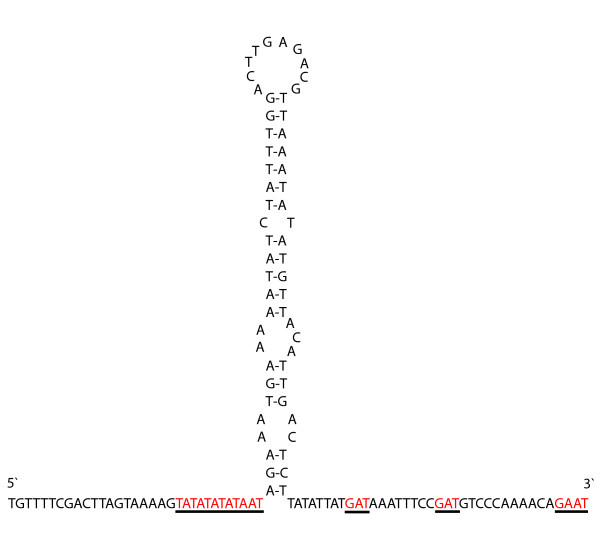
Stem-loop structure in the mitochondrial control region of *P. pearsei*. Underlined areas point out conserved motifs in 3'- and 5'-flanking sequences.

The generation of overlapping PCR fragments and subsequent sequencing demonstrated that the mitochondrial genome of *P. pearsei *is a typical circular DNA molecule with a length of 15099 bp [GenBank:EU024483]. All 37 genes usually present in bilaterian mitochondrial genomes have been identified (Fig. 1, Table 1). With the exception of five translocated tRNAs, gene order is similar to that of the horseshoe crab, *Limulus polyphemus*, which is considered to represent the putative ground pattern of the Arthropoda [38,39]. The tRNAs (*trnW*, *trnY*, *trnN*, *trnL*(CUN), *trnV*) changed their position to a new location between *trnM *and *nad2*. In this part of the genome there are five non-coding regions, which are ranging in size from 87 to 250 bp. The three largest non-coding regions are located between *rrnS *and *trnI *(250 bp), *trnY *and *trnN *(183 bp) and *trnV *and *nad2 *(169 bp). The shorter ones are situated between *trnI *and *trnQ *(100 bp) and *trnM *and *trnW *(87 bp). The longest non-coding region (250 bp) is flanked by *rrnSI *and *trn*, and based on its similarity to other arthropods [5,40,41] can be identified as the putative mitochondrial control region. Part of this region is capable of folding into a hairpin-like formation (Fig. 2) with a loop consisting of 10 nucleotides and a stem composed of 21 paired nucleotides (five mismatches). Furthermore conserved motifs occur in the flanking sequences around the stem-loop structure: a TATA motif appears in the 5'-flanking sequence whereas the motif GA(A)T is found in the 3'-flanking sequence (Fig. 2). Both motifs are also present in flanking sequences of other arthropods, e.g. in metastriate ticks [42], crustaceans [43], and insects and are presumed to have functional significance in transcription and/or replication [44]. The other larger non-coding regions do not bear similar hairpin-like structures.

**Table 1:** Genome organisation of *P. pearsei*. Complete circular mtDNA has a length of 15099 bp.

**Gene**	**Strand**	**Position**		**Length (nuc.)**	**CG-skew**	**Start-codon**	**Stop-codon**	**Intergenic nucleotides**
*cox1*	+	1-	1548	1548	0.277	ATG	TAA	-1
*cox2*	+	1548-	2218	671	0.414	ATG	TA	0
*trnK*	+	2219-	2289	71				-2
*trnD*	+	2288-	2350	63				0
*atp8*	+	2351-	2503	153	0.745	ATT	TAA	-7
*atp6*	+	2497-	3171	675	0.482	ATG	TAA	+3
*cox3*	+	3175-	3954	780	0.341	ATG	TAA	+2
*trnG*	+	3957-	4019	63				+3
*nad3*	+	4023-	4356	334	0.543	ATA	T	0
*trnA*	+	4357-	4421	65				-1
*trnR*	+	4421-	4482	62				+14
*trnS*(AGN)	+	4497-	4552	56				+3
*trnE*	+	4556-	4618	63				-2
*trnF*	-	4617-	4677	61				0
*Nad5*	-	4678-	6364	1687	-0.650	ATA	T	0
*trnH*	-	6365-	6425	61				+9
*Nad4*	-	6435-	7752	1318	-0.588	ATA	T	-4
*Nad4L*	-	7749-	8021	273	-0.872	ATG	TAG	+2
*trnT*	+	8024-	8085	62				0
*trnP*	-	8086-	8149	64				+8
*Nad6*	+	8158-	8589	432	0.587	ATA	TAA	+3
*Cob*	+	8593-	9697	1115	0.503	ATG	T	0
*trnS*(UCN)	+	9698-	9762	65				+3
*nad1*	-	9766-	10651	886	-0.588	ATA	T	+3
*trnL*(UUR)	-	10655-	10720	66				0
*rrnL*	-	10721-	11970	1250	-0.496			0
*rrnS*	-	11971-	12713	743	-0.471			+250
*trnI*	+	12964-	13020	57				+100
*trnQ*	-	13121-	13184	64				-4
*trnM*	+	13181-	13247	67				+87
*trnW*	+	13335-	13400	66				+10
*trnY*	-	13411-	13472	62				+183
*trnN*	+	13656-	13716	61				+17
*trnL*(CUN)	-	13734-	13800	67				+23
*trnV*	-	13824-	13888	65				+169
*nad2*	+	14058-	15020	963	0.556	ATT	TAA	+15
*trnC*	+	15036-	15096	61				+3

Besides the above mentioned regions only smaller non-coding regions of 2–23 bp are found in the mitochondrial genome. Gene overlaps occur between *cox1 *and *cox2 *(1 bp), *nad4 *and *nad4L *(4 bp), *atp6 *and *atp8 *(7 bp), and in four cases tRNA-genes are overlapping each other (Fig. 1, Table 1).

### Protein-coding genes and nucleotide composition

All of the 13 identified protein-coding genes begin with one of the common start codons for mtDNA ATG, ATA or ATT (Table 1). Out of these 13 protein-coding genes, six show incomplete stop codons (*cox2*, *cob *and *nad1*, *3*, *4*, *5*). In *cox2 *the stop codon is truncated and the gene terminates with TA, in the other five protein-coding genes only a single thymine serves as partial stop codon. Similar structural events have also been described for the mtDNA of other species where post-transcriptional polyadenylation completes a T or TA partial stop codon into a functional one [45].

The CG-skew (= (%C-%G)/(%C+%G)) of mitochondrial genes is a good indicator of the strand specific nucleotide frequency bias [46,47]. In *P. pearsei *the CG-skew is positive in all (+)strand encoded genes and negative in all (-)strand encoded genes (Table 1). We defined the (+)strand as the strand bearing the majority of coding sequence. The strand specific bias found in *P. pearsei *occurs in most other arthropods, while a reversal of that bias has been reported for only a few species [43,46,48-50]. Table 2 shows the CG-skews of third codon positions of *cox1 *for all chelicerates and outgroup taxa included in our phylogenetic analyses. We chose *cox1 *because this gene is found on (+)strand in all species examined. A reversal of CG-skew is seen in the two scorpions, in three Araneae (but not in *Heptathela*) and in the parasitiform mite *Varroa*.

**Table 2:** Nucleotide composition of third codon positions of the (+)strand encoded gene *cox1*, demonstrating strand bias in nucleotide frequencies within chelicerates.

**Species**	**Taxon**	**A**	**C**	**G**	**T**	**CG skew**	**Accession number**
*Lithobius forficatus*	Myriapoda	0.411	**0.200**	0.061	0.329	0.534	[GenBank:NC_002629]
*Daphnia pulex*	Crustacea	0.273	**0.211**	0.166	0.350	0.119	[GenBank:NC_000844]
*Penaeus monodon*	Crustacea	0.388	**0.133**	0.053	0.427	0.432	[GenBank:NC_002184]
*Limulus polyphemus*	Xiphosura	0.434	**0.229**	0.023	0.315	0.814	[GenBank:NC_003057]
*Oltacola gomezi*	Solifugae	0.407	**0.199**	0.055	0.339	0.569	[GenBank:EU024482]
*Centruroides limpidus*	Scorpiones	0.094	0.045	**0.264**	0.597	-0.709	[GenBank:NC_006896]
*Mesobuthus martensii*	Scorpiones	0.161	0.020	**0.309**	0.511	-0.881	[GenBank:NC_009738]
*Heptathela hangzhouensis*	Araneae	0.374	**0.096**	0.061	0.470	0.225	[GenBank:NC_005924]
*Ornithoctonus huwena*	Araneae	0.326	0.033	**0.166**	0.475	-0.667	[GenBank:NC_005925]
*Habronattus oregonensis*	Araneae	0.361	0.012	**0.101**	0.526	-0.792	[GenBank:NC_005942]
*Nephila clavata*	Araneae	0.379	0.016	**0.109**	0.496	-0.750	[GenBank:NC_008063]
*Pseudocellus pearsei*	Ricinulei	0.405	**0.281**	0.031	0.283	0.801	[GenBank:EU024483]
*Leptotrombidium akamushi*	Acariformes	0.356	**0.176**	0.084	0.384	0.354	[GenBank:NC_007601]
*Leptotrombidium deliense*	Acariformes	0.389	**0.123**	0.074	0.413	0.247	[GenBank:NC_007600]
*Leptotrombidium pallidum*	Acariformes	0.388	**0.114**	0.055	0.444	0.349	[GenBank:NC_007177]
*Amblyomma triguttatum*	Parasitiformes	0.392	**0.106**	0.043	0.459	0.421	[GenBank:NC_005963]
*Haemaphysalis flava*	Parasitiformes	0.433	**0.105**	0.043	0.419	0.421	[GenBank:NC_005292]
*Rhipicephalus sanguineus*	Parasitiformes	0.431	**0.086**	0.025	0.458	0.545	[GenBank:NC_002074]
*Ixodes hexagonus*	Parasitiformes	0.380	**0.197**	0.043	0.380	0.642	[GenBank:NC_002010]
*Ixodes holocyclus*	Parasitiformes	0.420	**0.093**	0.035	0.451	0.455	[GenBank:NC_005293]
*Ixodes persulcatus*	Parasitiformes	0.388	**0.103**	0.031	0.478	0.536	[GenBank:NC_004370]
*Ixodes uriae*	Parasitiformes	0.410	**0.173**	0.039	0.379	0.633	[GenBank:NC_006078]
*Carios capensis*	Parasitiformes	0.446	**0.185**	0.020	0.349	0.809	[GenBank:NC_005291]
*Ornithodoros moubata*	Parasitiformes	0.413	**0.164**	0.041	0.382	0.600	[GenBank:NC_004357]
*Ornithodoros porcinus*	Parasitiformes	0.378	**0.164**	0.064	0.394	0.436	[GenBank:NC_005820]
*Metaseiulus occidentalis*	Parasitiformes	0.400	**0.214**	0.037	0.349	0.708	[GenBank:NC_009093]
*Varroa destructor*	Parasitiformes	0.363	0.018	**0.062**	0.557	-0.560	[GenBank:NC_004454]

Bold numbers indicate higher values in comparison of G and C proportions. Only those taxa (24 chelicerate and 3 outgroup taxa) included in our phylogenetic analysis (Fig. 4) are listed.

### Secondary structure of transfer RNAs

**Figure 3:**
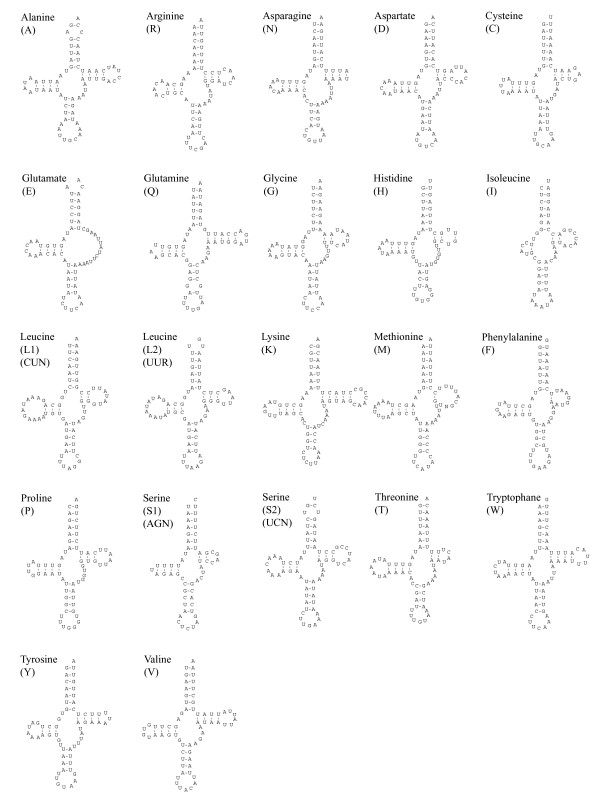
Putative secondary structures of mitochondrial tRNAs found in *P. pearsei*. All tRNAs can be folded into the usual cloverleaf secondary structure.

The mitochondrial genome of *P. pearsei *bears all of the 22 tRNAs commonly found in metazoan mtDNA (Fig. 1, Table 1). Except for tRNA-Glu, all tRNAs possess the typical cloverleaf secondary structure, though the TψC stem is shortened in several tRNAs (Fig. 3). The TψC-arm of tRNA-Glu is entirely absent. It is shortened to a single pair of nucleotides in tRNA-Met and tRNA-Phe and it is composed of just two paired bases in tRNA-Gly, tRNA-His, tRNA-Ile, tRNA-Leu (CUN), tRNA-Ser (AGN) and tRNA-Thr.

### Phylogenetic analysis

**Figure 4:**
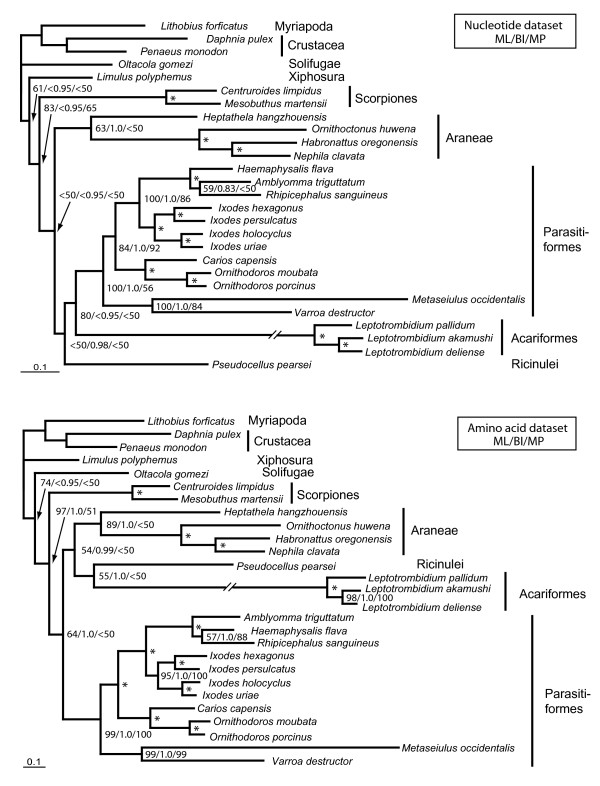
Phylogenetic trees of chelicerate relationships, inferred from nucleotide (upper) and amino acid (lower) datasets. All protein coding gene sequences were aligned and concatenated; ambiguously aligned regions were omitted by Gblocks. Trees were rooted with outgroup taxa (*Lithobius*, *Daphnia*, *Penaeus*). Topologies and branch lengths were taken from the best scoring trees of the maximum likelihood (ML) analyses. Numbers behind the branching points are percentages from ML bootstrapping (left), Bayesian posterior probabilities (BPP, middle) and maximum parsimony bootstrap percentages (MP, right). Stars indicate that values are 100 (ML), 1.0 (BI) and 100 (MP). See Table 2 for accession numbers.

We performed phylogenetic analyses with two different data sets: concatenated amino acid and concatenated nucleotide sequences (without third codon positions) from all protein-coding genes. Topologies with best likelihood scores from maximum likelihood (ML) analysis are shown (Fig. 4). Topologies of the 50% majority rule consensus trees from ML bootstrapping and Bayesian Inference (BI) differ only slightly from the best topologies of ML analysis: BI with the amino acid dataset resulted in a basal split between *Oltacola *and the remainder of chelicerates including *Limulus*, but that node is not supported by bootstrapping or BI; in ML bootstrapping with the nucleotide dataset there was no resolution between Ricinulei, Scorpiones, Araneae and Acari (ML bootstrap <50%). In all performed analyses, good support was found for monophyly of Scorpiones, Opisthothelae (= all Araneae except Mesothelae, here represented by *Heptathela*), *Ixodes*, *Leptotrombidium*, Ornithodorinae (*Carios *+ *Ornithodoros*), Ixodidae (*Ixodes *+ *Amblyomma *+ *Haemaphysalis *+ *Rhipicephalus*), Metastriata (*Amblyomma *+ *Haemaphysalis *+ *Rhipicephalus*), and Dermanyssina (*Metaseiulus *+ *Varroa*). Monophyly of Acari-Parasitiformes was also well supported by most analyses, except for the maximum parsimony (MP) bootstrapping (56%). Monophyly of Acari was recovered in the best topology of the nucleotide ML analysis and moderately supported by ML bootstrapping (80%), but not by BI or MP bootstrapping. Acari and Ricinulei form sister groups in the best ML topology in the analysis of the nucleotide alignment, but this clade found support only by BI (0.98), not by ML or MP bootstrapping. In contrast analyses of the amino acid dataset resulted in paraphyletic Acari, as Acariformes (*Leptotrombidium*) form a clade with Ricinulei, and both together form the sister group to Araneae. Both clades are well supported only by BI (Acariformes + Ricinulei: 1.0; Acariformes + Ricinulei + Araneae: 0.99).

Arachnid monophyly is only supported by ML analysis with the amino acid dataset (bootstrap: 74%). In all other analyses the three basal chelicerate branches – *Limulus *(Xiphosura), *Oltacola *(Solifugae), and the remainder of arachnids – are not well resolved. Monophyly of Arachnida excl. Solifugae (in this case Scorpiones + Araneae + Acari + Ricinulei) is well supported in ML and BI analyses of the amino acid alignment and weakly supported by ML analysis of the nucleotide alignment.

The classical, morphology based view of the phylogenetic position of Ricinulei is as the sister group to Acari [26-29]. Because our analysis does not resolve the basal relationships of arachnid orders well, it is not in conflict with actual morphology based analyses of arachnid interrelationships [27,36]. A sister group relation between Acari and Ricinulei is neither well supported, nor refuted by our results.

The phylogenetic analysis of mitochondrial genome data in chelicerate phylogenetics suffers from several problems. First, the currently incomplete taxon sampling (complete lack of data for Opiliones, Pseudoscorpiones, Palpigradi, Uropygi, and Amblypygi) highlights the preliminary nature of this analysis. While the problem of taxon sampling will be overcome in the near future, other problems lie in the nature of the sequence data itself. Several taxa show a reversed strand bias of nucleotide composition (Table 2), which complicates phylogenetic analyses [46,49]. This may strongly affect the phylogenetic position of Scorpiones in analyses with mitochondrial datsets [51]. Another problem is the heterogeneous substitution rate among arachnids. Our analysis demonstrates great variability in branch lengths, with very long branches for some Acari species (*Leptotrombidium, Varroa, Metaseiulus*), and very short branches, e.g. from *Limulus *and *Oltacola*.

### Mitochondrial gene order variation in Arachnida

**Figure 5:**
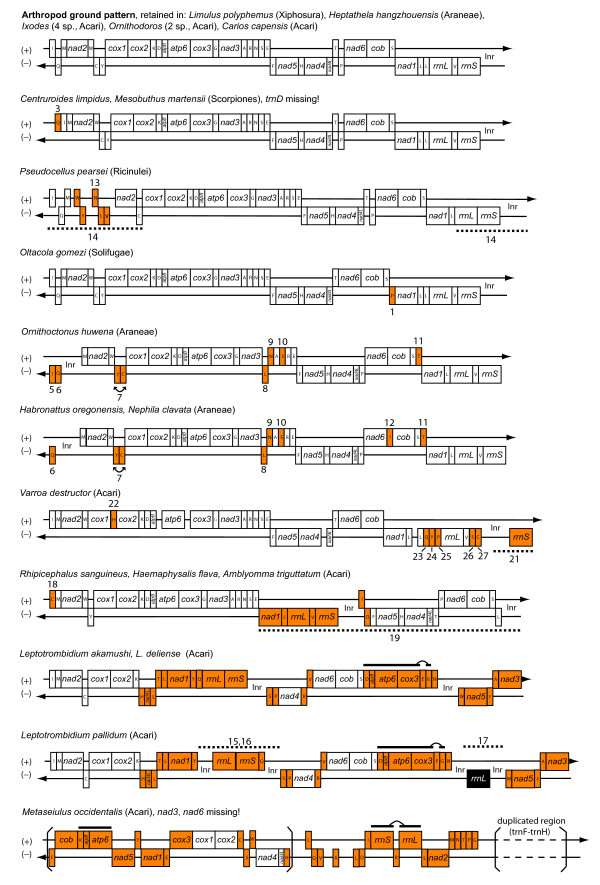
Changes in gene order in mitochondrial genomes of Arachnida compared to the putative ancestral arthropod gene order. Transfer RNA genes are labelled according to the one letter amino acid code. Genes marked white show the same relative position as in the arthropod ground pattern; genes marked orange have relative positions differing from the arthropod ground pattern; the gene marked black indicates a duplicated *rrnL *gene in *Leptotrombidium pallidum*. Horizontal lines above genes illustrate adjacent genes which were probably translocated together; dotted lines indicate regions where tandem duplication and random deletion events may have occurred; connected arrows show adjacent genes which have switched their position, making it difficult to assess which gene was translocated. Braces accentuate the duplicated regions in the mitochondrial genome of *Metaseiulus occidentalis*. lnr: large non-coding region, putative mitochondrial control region; other non-coding regions (> 50 bp) are illustrated by gaps between genes. Numbers refer to rearrangement events, compare Fig. 6. For GenBank accession numbers see Table 2.

**Figure 6:**
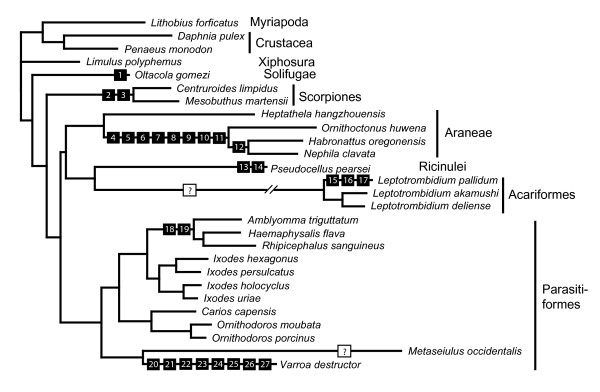
Hypothesized events leading to derived gene orders (black squares, compare Fig. 5) and strand bias reversal (red circles, compare Table 2) mapped on a topology based on the results of our phylogenetic analysis (compare Fig. 4, amino acid data set). **1**: Translocation of *trnP*; **2**: Reversal in nucleotide bias; **3**: Translocation of *trnQ*; **4**: Reversal in nucleotide bias; **5**: Translocation of *trnI*; **6**: Translocation of *trnQ*; **7**: Translocation of *trnY *or *trnC*; **8**: Translocation of *trnL*; **9**: Translocation of *trnN*; **10**: Translocation of *trnS*; **11**: Translocation of *trnT*; **12**: Translocation of *trnI*; **13**: Translocation of *trnN*; **14**: Tandem duplication of a gene block ranging from *trnL *to *trnY *followed by random deletion; **15, 16**: Two subsequent tandem duplications of a gene block ranging from *trnQ *to the large non-coding region followed by random deletion; **17**: Duplication and inversion of *rrnL *and a large non-coding region, probably by nonhomologous intergenome recombination (15–17 according to [59]); **18**: Translocation of *trnC*; **19**: Tandem duplication of a gene block ranging from *trnF *to *trnQ *followed by random deletion; **20**: Reversal in nucleotide bias; **21**: Tandem duplication involving the putative control region and *rrnS *followed by random deletion; **22**: Translocation of *trnH*; **23**: Translocation of *trnQ*; **24**: Translocation of *trnY*; **25**: Translocation of *trnP*; **26**: Translocation of *trnS*; **27**: Translocation of *trnC*; "**?**": Probably due to multiple rearrangements, the history of events leading to the highly derived gene order of *Leptotrombidium *and *Metaseiulus *cannot reliably be reconstructed.

Apart from five translocated tRNA-genes, the mitochondrial gene order of *P. pearsei *does not differ from the putative arthropod ground pattern (Figs. 5, 6). A minimum of two events is required to lead to the derived gene order of *P. pearsei*: a tandem duplication/random deletion event and a single tRNA gene transposition. Tandem duplication and random loss of genes is widely accepted as a mode of genome shuffling in mitochondria [52]. In the present case a segment ranging from *trnL *to *trnY *(ground pattern) can be inferred to be involved in such a duplication event. Subsequently the first copy may have lost *trnL*, *trnV*, *nad1*, and *trnC*, the second copy may have lost *rrnL*, *rrnS*, *trnI*, *trnQ*, *trnM*, *trnW*, and *trnY*, as well as the control region. Besides the control region (between *rrnS *and *trnI*) there are four larger non-coding regions (87–183 bp) present between *trnI *and *nad2 *(Fig. 1, Table 1). This is a further hint towards a tandem duplication random deletion event, although there are no apparent sequence homologies to any of the lost genes. In addition, *trnN *is located in a novel relative position which is best explained by the transposition of this single gene, the second inferred event.

The comparison of mitochondrial gene order from Arachnida reveals a great variation (Fig. 5). Only eight out of 23 species have retained the arthropod ground pattern as represented by *Limulus *[38]. We present a parsimonious scenario of gene order changes and mapped these events on a phylogenetic tree (Fig. 6). Three different modes leading to a change in gene order were assumed: (1) tandem duplication random deletion events [52], (2) inversions, and (3) transposition of single tRNAs. Although the latter mode is currently not well explained by a molecular model, we have assumed that single tRNA transpositions have occurred in all cases in which tandem duplication and random deletion seems to be an implausible explanation, e.g. when only one tRNA gene found a new position distant from its original position, or on the other strand. The tandem duplication random deletion model was proposed when more than one gene was involved and/or non-coding sequence was found between the genes involved. The tree we have used for mapping the events is the best scoring topology from the ML analysis of the amino acid alignment (Fig. 4, lower tree), because this analysis provided bootstrap support for most of the branches. However, regarding gene order changes there is also no character conflict (= homoplastic characters/events) with the other topology obtained from the nucleotide alignment.

None of the gene order characters is capable to resolve interordinal relationships of the taxa studied (Fig. 6). The arthropod ground pattern [38] is retained in some parasitiform mites (e.g. *Ixodes *[42], *Carios *and *Ornithodoros *[53]) (Figs. 5, 6), and in the spider *Heptathela *(Araneae: Mesothelae [41]). The mitochondrial genomes of Araneae-Opisthothelae (*Ornithoctonus *[41], *Nephila*, [GenBank:NC_008063], *Habronattus *[54]) share seven translocated tRNA genes. A subsequent translocation of *trnI *is found in *Nephila *and *Habronattus *[54]. The camel spider *Oltacola gomezi *(Solifugae) has a mitochondrial genome arrangement almost unaltered from the arthropod ground pattern [GenBank:EU024482], only *trnP *was translocated to a new relative position. The scorpions *Centruroides *[55] and *Mesobuthus *[56] share a translocated and inverted *trnQ *and both lack *trnD*.

Several independently derived mitochondrial gene orders are found in Acari. Metastriata (Parasitiformes: Ixodidae) show a derived gene order probably caused by a tandem duplication random deletion event and an additional tRNA translocation (*Rhipicephalus *[42], *Haemaphysalis *[53], *Amblyomma*, [GenBank:NC_005963]). The honeybee mite *Varroa *shows translocation of six tRNA genes [50]. In addition the reversed arrangement of *rrnS *and the control region in this species is hypothesized to be the result of a tandem duplication and random deletion event.

*Metaseiulus *(Parasitiformes: Mesostimata) possesses the most derived mitochondrial gene order among arachnids, probably due to multiple rearrangements [57]. This genome is the largest (25 kb) within the Chelicerata due to the presence of duplicate and triplicate regions. The duplicate region contains 18 genes plus a copy of the control region and partial *trnL*(UUR) sequence while the triplicate region comprises only the control region sequence and partial *trnL*(UUR) sequence (Fig. 5). The mitochondrial genome of *Metaseiulus *is also remarkable for the absence of *nad3 *and *nad6*, because no other chelicerate is known to have lost any of the protein coding genes. Due to the magnitude of these changes, it is difficult to reliably reconstruct the events leading to the gene order found in *Metaseiulus *which is unique amongst arachnids.

The same is true for the genus *Leptotrombidium *(Acariformes) [58,59]. The three species share a common, derived arrangement with secondary rearrangements in *L. pallidum*. The secondarily derived gene order of *L. pallidum *(translocation of *trnQ*, duplication of *rrnL *and the presence of four large noncoding regions) is considered to have evolved by a combination of tandem duplication with random deletion plus intergenomic recombination between several genes with subsequent gene conversion [59].

It is apparent that the two taxa showing the most complicated genome rearrangement history (*Leptotrombidium *and *Metaseiulus*) exhibit also the longest branches (= highest substitution rates) in phylogenetic analysis (Fig. 4, 6).

### Reversals of strand bias in nucleotide frequency

A reversal of nucleotide strand bias was detected in six species (Table 2), most probably due to three independent events: (1) in the *Varroa *mite [50], (2) in Scorpiones and (3) in Araneae-Opisthothelae (*Ornithoctonus *[41], *Nephila*, [GenBank:NC_008063], *Habronattus *[54]). These changes are also mapped on the tree in Fig. 6 (characters 2, 4, and 20). The comparatively low substitution rate in the mitochondrial genome of *Heptathela *(Araneae: Mesothelae; Fig. 6) argues against a re-reversal of nucleotide bias within Araneae and suggests that the reversal is in fact a synapomorphy of the Opisthothelae (Fig. 6).

## Conclusion

The first complete mitochondrial sequence of a hooded tickspider (*P. pearsei*, Ricinulei, Arachnida) reveals a typical circular duplex DNA molecule with a compact gene organisation as found in other bilaterians. In comparison to the putative arthropod ground pattern we observed a derived gene order with five tRNA genes found in different relative positions compared to the gene order of *Limulus*. Probably two events led to the derived gene order of *Pseudocellus*: (1) a duplication and random deletion event may be responsible for the translocation of four of these tRNA genes and (2) an additional translocation of the *trnN*. The putative mitochondrial control region is situated in the same position as in other arthropod mitochondrial genomes. Part of the putative control region can be folded into a characteristic stem-loop structure with conserved flanking sequences as found in other arthropods. All tRNAs, except tRNA-Glu, can be folded into a typical cloverleaf secondary structure.

Aligments of nucleotide and amino acid sequences from mitochondrial protein-coding genes were used in a phylogenetic analysis of arachnid relationships. In the best scoring topologies from ML analyses, Ricinulei appear as sister taxon to Acari (nucleotide alignment) or Acariformes (amino acid alignment), but in both cases without sufficient support from bootstrapping or Bayesian inference. Within the Acari, monophyly of Parasitiformes is well supported, while Acariformes are represented by only a single genus (*Leptotrombidium*). Monophyly of Acari was only recovered in the analysis of the nucleotide alignment and found strong support by BI and only weak support by ML/MP bootstrapping.

Because of highly divergent substitution rates amongst arachnid species, phylogenetic analyses may be generally biased due to long-branch attraction. Another complicating factor is the reversal of nucleotide bias, which has occurred independently in the arachnid clades Scorpiones, Acari (*Varroa*) and Araneae (Opisthothelae). Due to the lack of support for interordinal relations within Arachnida, the phylogenetic analysis of mitochondrial genome data reveals no strong conflict with recent morphological analyses.

Investigation of mitochondrial gene rearrangements across the range of taxa for which complete mitochondrial genomes are available on GenBank, reveals high variability in gene order within arachnids. Among arachnids, 15 of the 23 species investigated have derived features in gene order. When mapped onto the tree, the hypotheses of events leading to gene order changes are not in conflict with our phylogenetic analyses of sequence data. None of the hypothesized events is useful as a phylogenetic character to resolve interordinal relationships within arachnids, but some of these characters are promising in analyses of intraordinal relationships. Especially in Acari the comparison of mitochondrial genomes from a larger taxon sampling would be promising for the detection of gene order changes, which will be valuable in a phylogenetic analysis. Altogether, further mitogenomic data of a broader taxon sampling are necessary, especially from Opiliones, Pseudoscorpiones, Palpigradi, Uropygi and Amblypygi.

## Methods

### Samples and DNA extraction

*P. pearsei *specimens were collected by Gerd Alberti in the Gruta Sabac-Ha, near Merida, province of Yucatán, México (20°10'18"N, 89°16'03"W). DNA extraction was done with two legs obtained from a freshly killed animal. We used "DNeasy blood and tissue kit" (Qiagen, Hilden, Germany) according to the manufacturers protocol, except for reduction of the elution volume to 50 μl.

### PCR and sequencing

Initial PCRs were done with a published primer set designed for crustacean mitochondrial genomes [60]. All PCRs were performed on an Eppendorf Mastercycler and Mastercycler gradient. The Eppendorf 5-prime-Taq kit (Eppendorf, Germany) was used in 50 μl volumes (5 μl buffer; 1 μl dNTP mix, 10 μM; 0.25 μl Taq polymerase; 1 μl template DNA, 40,75 μl water, 1 μl primer mix, 10 μM each). PCR conditions were: initial denaturation (94°C, 1 min), 40 cycles of denaturation (94°C, 30 sec), annealing (50°C, 30 sec), and elongation (68°C, 1 min), followed by a final elongation step (68°C, 1 min). Successful PCR amplification was obtained with six primer pairs: S8(*cox1*, about 650 bp), S10(*cox1*, about 1050 bp), S15(*cox2-atp6*, about 830 bp), S20(*cox3-trnF*, about 950 bp), S37(*nd4l-cob*, about 1600 bp), and S46(*rrnL-rrnS*, about 1000 bp), see [60] for primer sequences. PCR products were visualized on 1% agarose gels. Before sequencing PCR products were purified using the Bluematrix DNA purification kit (EURx, Gdansk, Poland). Except for S8 and S10 there was no overlap between these initial sequences. Species specific primer pairs were designed to bridge the five gaps between these initial sequences (see Table 3 for primer information). Long PCRs were performed with Takara LA Taq kit (Takara) in 50 μl volumes (5 μl Buffer, Mg+; 8 μl dNTP mix, 2.5 μM; 1 μl DNA; 1 μl primer mix, 10 μM each; 0.5 μl Takara LA Taq; 32 μl water). PCR conditions were: initial denaturation (94°C, 1 min), 40 cycles of denaturation (94°C, 30 sec), annealing (primer specific temperature see Table 3, 1 min), and elongation (68°C, 3 min), followed by a final elongation step (68°C, 2 min). Long PCR products were sequenced by primer walking. All sequencing was performed on a CEQ 8000 capillary sequencer (Beckmann-Coulter) using CEQ DCTS kits (Beckmann-Coulter) with 10 μl reaction volumes (4 μl DCTS master mix, 1 μl primer, 10 μM, 1–5 μl DNA, 0–4 μl water). Sequencing reaction was done with 30 cycles of denaturation (94°C, 30 sec), annealing (primer specific temperature, 30 sec) and elongation (60°C, 2 min).

**Table 3:** Species specific PCR primer pairs used for amplification of fragments from the mitochondrial genome of *Pseudocellus pearsei*.

***Primer name***	***Nucleotide sequence (5'-3')***	***Annealing temp.***
Rici-12s-co1	CCACATTACAACATAGTAACTCATTTC	56°C
Rici-co1-12s	AAGTTCACCCTGTTCCTGCTC	56°C
Rici-co1-co2	ATTACGTTGTAGCACACTTCCAC	56°C
Rici-co2-co1	AGATGGTAATGTTAGTAATATTTGGTG	56°C
Rici-co2-co3	GTAACATCAACTCTAGCACTAACAC	50°C
Rici-co3-co2	GTGTCGTGGAAATTGGGA	50°C
Rici-co3-cob	CTATCAATCTAACCCAACAAAAAAG	54°C
Rici-cob-co3	GTCAGAAGATAGTTTATTGGAATATTGGC	54°C
Rici-cob-12s	CCACCATTAACACCCAAAGCC	56°C
Rici-12s-cob	TTGGATTTAATAGTAAGGAAGTATTAGATAGG	56°C

### Sequence assemblage and annotation

Primary sequence analysis was performed with the CEQ software (quality check). Sequence assemblage was done with Bioedit 7.0.1 [61]. Protein-coding and ribosomal RNA genes and gene boundaries were identified by BLAST search and in comparison with alignments from other chelicerate species. Genomic position and secondary structure from 20 out of 22 transfer RNAs were identified by tRNA-scan SE [62], the remaining two by eye inspection of the regions under suspect. Sequence data was deposited at NCBI database [GenBank:EU024483].

### Phylogenetic analysis

Alignments from all protein-coding genes were used in phylogenetic analysis. Alignments of amino acid sequences and nucleotide sequences were performed with ClustalW [63], as implemented in Bioedit 7.0.1 [61]. Nucleotide sequences were translated to amino acid sequence prior to the multiple alignment and translated back afterwards (making use of the "toggle" function of Bioedit). Sequence data was obtained from 24 chelicerate species and three outgroup taxa (for a list of taxa with GenBank accession numbers see Table 2). Ambiguously aligned regions were omitted using Gblocks ver. 0.91b [64], using default settings, except for changing "allowed gap positions" to "with half". The final amino acid alignment consisted of 2786 amino acids. For the nucleotide dataset the "codons" option was used, so that only complete codons (not single nucleotides) were omitted from the alignment by Gblocks. A saturation analysis [65] was performed with DAMBE 4.2.13 [66] using subsets of the nucleotide dataset representing first, second and third codon positions. Due to significant saturation, third codon positions were omitted from the final nucleotide alignment (6490 nucleotides).

Maximum parsimony (MP) analysis was performed with PAUP* ver. 4.0b10 [67], 1,000 bootstrap replicates were performed, each with 10 replicates with random addition of taxa. Maximum likelihood (ML) analysis was done with Treefinder [68]. Model selection was done with ProtTest ver. 1.3 [69]. According to the Akaike information criterion, the mtArt+G+I model was optimal for ML analysis. The tree with the best likelihood value was chosen out of 10 independent analyses with random taxon order. Bootstrapping was performed with 100 pseudoreplicates. Bayesian inference (BI) with MrBayes ver. 3.1.2 [70] was done with the model mtRev+G+I, as the mtArt model is not implemented in the current version of MrBayes. Eight chains ran for 1,000,000 generations, while tree sampling was done every 1,000 generations; we examined likelihood values of the sampled trees and decided to omit the first 100 trees as burn-in, while the remaining 900 were used to calculate Bayesian posterior probabilities (BPP).

## Abbreviations

A, adenine; *atp6 *and *8*, genes encoding ATPase subunit 6 and 8; BI, Bayesian inference; bp, base pairs; BPP, Bayesian posterior probability; *cox1*-*3*, genes encoding cytochrome oxidase subunits I-III; *cob*, gene encoding cytochrome b; C, cytosine; G, guanine; ML, maximum likelihood; mtDNA, mitochondrial DNA; *nad1*-*6 *and *nad4L*, genes encoding NADH dehydrogenase subunits 1–6 and 4L; PCR, polymerase chain reaction; rDNA, ribosomal DNA; rRNA, ribosomal RNA; *rrnL*, large (16S) rRNA subunit (gene); *rrnS*, small (12S) rRNA subunit (gene); T, thymine; TDRD, tandem duplication and random deletion; tRNA-Xxx (where Xxx is replaced by three letter amino acid code of the corresponding amino acid), transfer RNA; *trnX *(where *X *is replaced by one letter amino acid code of the corresponding amino acid), tRNA gene; TψC-arm, T-loop and T-stem of a tRNA secondary structure.

## Competing interests

The author(s) declares that there are no competing interests.

## Authors' contributions

GT sampled and determined animals and isolated DNA. KF, GT and LP performed PCRs, KF and LP did all sequencing, sequence analysis and annotation. KF, AB and LP performed phylogenetic analyses. KF and LP wrote the manuscript.
